# Advances in Regulatory Strategies of Differentiating Stem Cells towards Keratocytes

**DOI:** 10.1155/2022/5403995

**Published:** 2022-01-31

**Authors:** Aini Zhang, Wei Zhang, Ludvig J. Backman, Jialin Chen

**Affiliations:** ^1^School of Medicine, Southeast University, 210009 Nanjing, China; ^2^Jiangsu Key Laboratory for Biomaterials and Devices, Southeast University, 210096 Nanjing, China; ^3^China Orthopedic Regenerative Medicine Group (CORMed), China; ^4^Department of Integrative Medical Biology, Anatomy, Umeå University, SE-901 87 Umeå, Sweden; ^5^Department of Community Medicine and Rehabilitation, Physiotherapy, Umeå University, SE-901 87 Umeå, Sweden

## Abstract

Corneal injury is a commonly encountered clinical problem which led to vision loss and impairment that affects millions of people worldwide. Currently, the available treatment in clinical practice is corneal transplantation, which is limited by the accessibility of donors. Corneal tissue engineering appears to be a promising alternative for corneal repair. However, current experimental strategies of corneal tissue engineering are insufficient due to inadequate differentiation of stem cell into keratocytes and thus cannot be applied in clinical practice. In this review, we aim to clarify the role and effectiveness of both biochemical factors, physical regulation, and the combination of both to induce stem cells to differentiate into keratocytes. We will also propose novel perspectives of differentiation strategy that may help to improve the efficiency of corneal tissue engineering.

## 1. Introduction

In the eye, the cornea is the outermost structure—a highly organized and specialized transparent tissue that plays a vital role in both the refraction of light onto the retina and protecting the eye from infectious agents. When the cornea is injured, activated keratocytes in the corneal stroma will transform into fibroblasts and myofibroblasts and subsequently migrate to the wound for tissue remodeling, including alignment of collagen fibrils. However, under pathophysiological conditions, a fibrotic process may occur, resulting in corneal scar formation, including misaligned collagen fibrils. The pathophysiology of corneal scar formation is still poorly understood, thus the current lack of treatment to restore the structure of the collagen fibrils and regain vision. Corneal transplantation is presently the only available curative treatment for corneal scars, but even in many developed countries, it is difficult to perform transplantations due to lack of donors. Therefore, new therapeutic methods to treat corneal scar formation are warranted.

Recently, much research focus has been on corneal tissue engineering [1–7]. The research has mainly focused on attempts to create artificial corneal constructs to implant clinically in patients, which could potentially solve the problem of limited donors. In these attempts, stem cells are commonly used and differentiated into corneal keratocytes. However, the utilization of stem cells in corneal tissue engineering is not without challenges since the appropriate microenvironment is crucial to achieve differentiation of stem cells specifically into keratocytes.

In this review, we aim to clarify the role and effectiveness of both biochemical factors, physical regulation, and the combination of both to induce stem cells to differentiate into keratocyte. In addition, we will propose novel perspectives of differentiation strategy that may help to improve the efficiency of corneal tissue engineering.

## 2. Cornea and Corneal Injury

**Figure 1:**
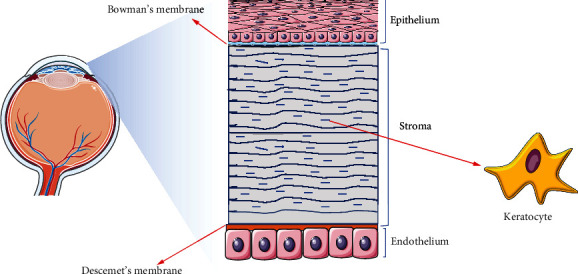
Schematic structure of the cornea. The epithelial layer is the outermost part of the corneal tissue, which sits on Bowman's membrane. The stroma layer is the middle part of the corneal tissue which accounts for 80%-85% of the cornea's thickness and consists of mainly keratocytes. The endothelium layer is the innermost part of the corneal tissue and is connected to the stroma layer by Descemet's membrane.

The cornea is comprised of five main layers from front to back: epithelium layer, Browman's membrane, stroma layer, Descemet's membrane, and endothelium layer (Figure 1) [8]. The avascular stroma accounts for 80%-85% of the cornea's thickness, which is around 0.4 mm at the center and gradually increases in thickness towards the periphery [9]. It consists of keratocytes, collagens (mainly collagen type I and type V), glycosaminoglycans (GAGs), and proteoglycans (PGs) [8]. Maintenance of transparency and mechanical characteristics in the stroma is highly dependent on its orthogonal orientation of collagen fibers, arranged into bundles called lamellae, with a thickness of 2 *μ*m-200 *μ*m. In between the lamellae, the sparsely scattered collagen-producing keratocytes are found [10]. The keratocytes are normally considered quiescent and exhibit a dendritic morphology with processes extending and interacting with neighboring cells and express cluster of differentiation 34 (CD34) and aldehyde dehydrogenase 3 family (ALDH3A1). Keratocytes produce various important PGs including keratocan (KERA), lumican (LUM), mimecan, decorin, and biglycan [8]. These PGs consist of different GAGs such as keratan sulfate, chondroitin sulfate, and dermatan sulfate [9]. Furthermore, keratocytes are responsible for the synthesis of collagen molecules and matrix metalloporteases (MMPs) which is of significance in maintaining the homeostasis of corneal stroma [9].

Corneal injury is one of the leading causes of blindness, affecting millions of people worldwide [11]. When the cornea is injured, activated corneal stroma keratocytes will no longer be able to perform its physiological functions but will instead transform into fibroblasts and myofibroblasts that under pathophysiological conditions may result in scar formation and blindness. According to the survey of WHO, there are over half a million people worldwide who are suffering from blindness caused by eye injuries and around 48% of them were specifically caused by corneal injuries [12, 13]. Currently, the main therapeutic option for corneal impairment is corneal transplantation. The demand of corneal transplantation has gradually increased, but there is a lack of accessible cornea for transplantation. It is reported that around 12.7 million individuals are waiting for corneal transplantation but only 1/7 can be transplanted, mainly due to lack of donors [8, 14]. According to a survey done in 2016, the median waiting time was 6.5 months before a suitable cornea was donated for transplantation while in many countries, patients were unable to receive a transplantation and became blind [15]. Therefore, there is an urgent need of new strategies to tackle the shortage of corneal donors for transplantation.

## 3. Corneal Tissue Engineering

Tissue engineering is an interdisciplinary research field that is aimed at restoring or regenerating the impaired tissues *in vivo*. More specifically, cells are isolated from tissue and expanded in culture *in vitro*. Biomaterials (scaffold) and/or biochemical factors are combined with the cells and subsequently implanted to the body to allow regeneration of the defected tissues or organs to improve the quality of patients' life [16, 17]. Up to now, tissue engineering has already been applied in the regeneration of miscellaneous tissues/organs such as the liver [18–20], spinal cord [19, 21], skin [22–24], cartilage [25], and blood vessels [26]. In the field of corneal tissue engineering, researchers have transplanted decellularized human corneal lamina with autologous adipose-derived MSCs (ASCs) into patients with corneal defects to evaluate its safety, tolerability, and preliminary efficacy. In addition to this, ex vivo cultivated human corneal stromal stem cells (CSSCs) have been transplanted into recruited patients to treat corneal blindness. These clinical trials to achieve regeneration of the human cornea and the restoration of vision have been summarized in Table 1. Since clinical trials show promising results, it is tempting to believe that tissue engineering can be used to replace impaired cornea to tackle the shortage of corneal donors.

## 4. Cells Used in Tissue Engineering of Corneal Stroma

In recent years, there have been large number of studies on corneal tissue engineering. The selection of appropriate cells is important for successful tissue engineering and is considered the first core factor in the field of tissue engineering. Even though keratocytes are the main cell type in the normal cornea, it is not a suitable cell type for corneal tissue engineering because of the difficulty to culture keratocytes *in vitro*. When keratocytes are cultured in medium containing serum, they quickly lose their dendritic morphology, decrease the expression of specific keratocyte markers, and instead transform into a fibroblast and myofibroblast phenotype [27] in a similar manner as in injured cornea. To prevent the phenotype drift of cultured keratocytes, serum-free medium has been developed. Although keratocyte phenotype can be maintained by culturing them in serum-free medium, their low proliferation rate makes it hard to obtain enough cells required for tissue engineering [27]. Some researchers have generated a cell culture system which can both ensure the expansion of human keratocytes and the preservation of their dendritic morphology by culturing them on human amniotic membrane [27]. However, the procedure is complex and requires expensive growth factors [27, 28]. Therefore, based on the arguments raised above, keratocytes are not suitable cells for corneal stroma tissue engineering.

Stem cells, which obtain outstanding proliferative capacity and the potential to differentiate into keratocytes, have been widely used in corneal stroma tissue engineering. The main types of stem cells used are CSSCs [29–31], embryonic stem cells (ESCs) [5, 32–35], and mesenchymal-derived stem cells (MSCs) [27, 36–38]. MSCs are mainly divided into two categories: bone marrow-derived MSCs (BMSCs) and ASCs. CSSCs could be isolated from the limbal region of human corneas, using the neutral protease dispase. Unlike keratocytes, CSSCs are able to undergo extensive expansion *in vitro* without losing the ability to differentiate into keratocyte phenotype [29]. CSSCs embedded in compressed collagen gel has been injected into the injured cornea of mouse which resulted in successful regeneration without scar formation after 2 weeks [39]. Compared with the CSSCs, MSCs are easier to obtain from either bone marrow (BMSC) or adipose tissues (ASC), and their self-renewal ability has been proven. Liu et al. injected BMSCs into the kera^−/−^ or lum^−/−^ mouse model. It was found that BMSCs were able to survive in the corneal stroma and to differentiate into a keratocyte phenotype [36]. However, MSCs have the potential to differentiate into multiple cell types such as cardiomyocytes and vascular endothelial cells, thus making a specific differentiation into keratocytes a challenge [27, 29]. ESCs, which derived from the inner cell mass of human blastocyst, appear to have an unlimited lifespan and the potential to differentiate into any somatic cell type [32]. However, ESCs need to be cocultured with other cells, such as the mouse fibroblast line PA6, to differentiate into the neural crest-lineage, that is considered to be the origin of keratocytes, before being differentiated into keratocyte phenotype [32]. Yet, the ethical aspect of using ESCs hampers its application in corneal tissue engineering [40]. As a result of a profound exploration of the corneal keratocytes, researchers found that dental stem cells (DSC) and keratocytes share the same origin from the neural crest lineage. In addition, DSCs are easily accessible and share similar proteoglycan secretion profile as keratocytes which make them a promising cell type for corneal tissue engineering [41, 42]. As an example, dental pulp stem cells (DPSCs) injected into mouse corneal stroma was discovered to form a stromal extracellular matrix (ECM) without rejection, thus suggesting that it may serve as a possible choice of cells for corneal regeneration [43]. Although all the above-mentioned stem cells are promising for corneal tissue engineering and regeneration, they have great uncertainty since they harbor multipotent differentiation potential. Therefore, many studies have tried to improve the efficiency of cell differentiation towards keratocytes by using various strategies of regulation.

## 5. Current Strategies for Inducing Differentiation of Stem Cells into Keratocytes

Extensive studies have shown that the microenvironment plays a significant role in modulating the differentiation fate of stem cells, which includes stem cells in the bone marrow [44], skin [45], intestine [46], brain [47], spinal cords [48], and others [49, 50]. The microenvironment includes both the physical (stiffness, stress or strain relaxation, etc.) and biochemical factors (growth factors or cell adhesion molecules, etc.) [51]. As an example, in osteogenesis, it was found that both the cell-cell contact (transmission of growth factors, i.e., biomechanical factors) and the substrate stiffness (physical environment) played important roles in the differentiation of MSCs towards osteoblasts and are therefore considered important in regeneration of bone [52]. The sophisticated 3D ECM of corneal stroma consists of highly aligned collagen layers and multiple growth factors including insulin-like growth factors (IGF), transforming growth factor beta (TGF-*β*), and fibroblast growth fator-2 (FGF-2) [53, 54]. The cells in the corneal stroma also receive various kinds of physical stimulations (the stiffness of ECM, local topography, and stress), which is crucial for the developmental morphogenesis, reaction to fluctuating intraocular pressure, and wound healing process of keratocytes [55].

**Figure 2:**
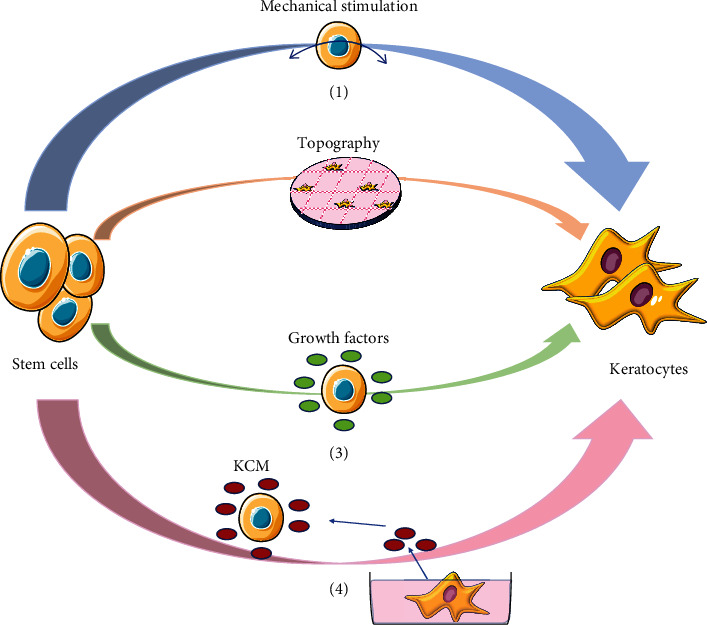
Current strategies for directing stem cells into keratocytes. (1) Dome-shaped mechanical stimulation; (2) topography; (3) growth factors; (4) keratocyte-conditioned medium (KCM).

Inspired by the microenvironment, current methods used by researchers to differentiate stem cells towards keratocytes include biochemical stimulation (culture media, growth factors, etc.), physical regulation (dome-shaped mechanical stimulation and topography), and the systematic regulation (the combination of both the physical and biochemical stimulations) (Figure 2).

### 5.1. Biochemical Regulation

Previously, biochemical induction of various categories of stem cells to differentiate into keratocytes has been extensively studied [1, 2, 7, 27, 29, 30, 36, 38, 41, 53, 56–58] and proven to have positive effects. Most of these studies showed significant upregulation of specific keratocyte gene expressions and some of the studies even obtained differentiated cells with keratocyte-like dendritic morphology [57] (Table 2).

As earlier mentioned, even though keratocytes can be harvested from the cornea, it is difficult to sustain their morphology and functions *in vitro* [59]. Some studies tried to achieve proliferation and the maintenance of keratocyte phenotype by culturing them on animal corneal tissue [36] or on amniotic membrane (AM) [28] which has a similar microenvironment as the cornea. Park et al. previously obtain cornea-like epithelial cells from MSCs using corneal epithelial cell-conditioned medium, which inspired them to generate a similar keratocyte-conditioned medium (KCM) in order to stimulate the differentiation of human MSCs (hMSCs) towards keratocytes [27]. KCM is a medium that is believed to contain specific biochemical factors that mimic the keratocyte microenvironment *in vivo* and thus believed to stimulate the differentiation of human stem cells into keratocytes. When hMSCs were cultured in KCM on plastic dishes, the gene expression of keratocyte markers (LUM and ALDH1A1) were increased and the expression of *α*-smooth muscle actin (*α*-SMA) was decreased [27]. This specific study is important as it is the first study to prove that it is possible to stimulate stem cell differentiation into keratocytes *in vitro* and it also emphasized the possibility of using biochemical factors for keratocyte differentiation. However, it was unknown which biochemical factor/factors in KCM played a crucial role in the keratocyte differentiation [27].

To address this question, Park et al. cultured human BMSCs with KCM supplemented with various concentrations of insulin-like growth factor binding protein 2 (IGFBP2) for 24 hours. They found increased expression of keratocyte markers (KERA and ALDH1A1) and decreased expression of myofibroblasts marker *α*-SMA, with the greatest effect at supplementation of 500 ng/ml of IGFBP2 [38]. In addition to this, Kafarnik et al. found that human CSSCs exposed to 10 ng/ml FGF-2 presented a keratocyte-like morphology and expression profile (increased expression of keratocyte markers, decreased expression of myofibroblastic markers and stem cell markers) [57]. Similarly, Wu et al. treated CSSCs with either 0.1 ng/ml TGF-*β*3, 10 ng/ml FGF-2, or a combination of both factors for 9 weeks. It was found that FGF-2 and TGF-*β*3 had a synergistic effect on keratocyte differentiation [53] and resulted in superior cell viability as compared to the cells that had only been added with TGF-*β*3 or FGF-2 and the cells exhibited a dendritic morphology similar to keratocytes. Additionally, the expression of keratocyte marker ALDH3A1 and carbohydrate sulfotransferase 6 (CHST6) was significantly increased and the expression of myofibroblast (*α*-SMA) was inhibited. Simultaneous stimulation of FGF-2 and TGF-*β*3 on human CSSCs also induced multilayered lamellae with orthogonally oriented collagen fibrils, mimicking the human corneal stromal tissue, which was not seen when FGF-2 or TGF-*β*3 were given separately [53]. Presently, there are mainly two typical types of keratocyte differentiation media (KDM). One is composed of advanced DMEM, ascorbate-2-phosphate (A2-P), FGF-2, and TGF-*β*3 [41]. The other differentiation media is mainly based on advanced DMEM, FGF-2, and A2-P [2]. This implies that the composition and concentration of growth factors in the different medium could be slightly different in different studies even though the main components of KDM are the same. The induction of keratocyte differentiation by KDM has been confirmed in various kinds of stem cells, including ASCs [1], BMSCs [36], CSSCs, [30], induced pluripotent stem cells (iPSC) [33, 60], and periodontal ligament stem cells (PDLSCs) [42]. According to those studies, KDM had a positive influence on stem cell differentiation into keratocytes which is supported by a dramatic increase of gene expressions of keratocyte markers (including KERA and LUM) and the deposition of orthogonally oriented collagens [3].

However, it is noted that keratocyte-like cells derived from differentiated stem cell are still different from the natural keratocytes in both the level of gene expression and the expression profile and the cell morphology [7]. New strategies are warranted to be developed to improve the efficiency of keratocyte differentiation.

### 5.2. Physical Regulation

In order to obtain superior keratocyte morphology and function from stem cells, attention has been focused not only on the biochemical stimuli but also to the physical environment of the corneal stroma. As mentioned, physical stimulations such as local topography and stress play significant roles in the regulation of keratocyte behavior *in vivo*. Therefore, physical regulation such as mechanical stimulation has been considered to be a possible way to stimulate stem cell differentiation into keratocytes. The positive influence of mechanical stimulation on stem cell differentiation has been widely studied in other tissues and cell types, including chondrogenesis [61] and differentiation into tenocytes [62]. Unlike other tissues, corneal stroma is under dome-shaped strain. Therefore, Chen et al. applied a dome-shaped static mechanical stimulation, mimicking the *in vivo* cornea, and discovered that it was able to upregulate the expression of ALDH3A1, CD34, LUM, COL I, and COL V in PDLSCs [3]. The dome-shaped mechanical stimulation also had a synergistic effect in combination with KDM. The results of this study suggest that physical strategies are a promising method in the regulation of keratocyte differentiation.

Additionally, topographical cues in the microenvironment are essential in the regulation of cell behaviors during both the physiological and pathological conditions. The arrangement and the length of the ECM components are able to modulate a broad range of cell behaviors like adhesion, morphology, and differentiation [55]. In the corneal stroma, the highly organized environment composed of a unique arrangement of collagen lamellar has been proven to be significant in obtaining keratocyte morphology and offers a topographical cue to the differentiation of keratocytes [63, 64]. Teixeira et al. cultured human keratocytes on silicone substrate containing grooves and ridges, ranging from 70 to 2000 nm which strongly align the keratocytes in the direction of the anisotropic patterns. Generation of focal adhesions and stress fibers by keratocytes was reduced on 70 nm-wide ridges when compared to micron-size patterns or smooth substrates. This observation indicated that the keratocytes are sensitive to the anisotropic topographic stimuli and that suitable substrate topographies are able to affect the behavior of keratocytes such as the keratocyte-myofibroblast transdifferentiation [63]. Moreover, hCSSCs were cultured on both aligned and random polyurea fibers in KDM to explore the role of topography in inducing an ECM secretion profile similar to that of the native corneal stroma. Wu et al. found that hCSSCs differentiated into keratocytes seeded on aligned substrates secreted more ECM, similar to the native corneal stroma [64]. Thus, the physical environment is essential for the maintenance of both the function and the behavior of keratocytes and offers a clue for the differentiation of stem cells towards functional keratocytes.

### 5.3. Systematic Regulation

Since neither biochemical regulation nor physical regulation alone is able to achieve the desired effect, researchers tend to combine the two approaches to achieve more sufficient differentiation result (Table 3). Yam et al. cultured PDLSCs in a 3D pellet model (cells were suspended in a spherical shape) and subsequently induced differentiation with KDM [6]. The pellet model is considered to increase cell-to-cell interactions (mainly cadherin-containing and connexin-containing junctions) [31] and was accepted as an appropriate model since it promotes production of total collagen and expression of KERA, the major proteoglycan in corneal stroma [65]. In Yam et al.'s study, they used the pellet model with KDM and more specifically CSK (corneal stroma keratocyte) induction media, which is mainly based on FGF-2, TGF-*β*3, and DMEM/F12. They found that the stimulation upregulated the expression of keratocyte-specific markers (ALDH3A1, KERA, LUM, CHST6, B3GNT7, and Col8A2) and downregulated the expression of genes related to fibrosis and other lineages [6]. Some researchers also induced totipotent stem cells (human iPSCs [33] and ESCs [34]) into keratocytes by using the method of combining both the physical and the biochemical strategies. As both iPSCs and ESCs usually are difficult to differentiate into specialized mature cells, it is performed in a two-step procedure by first inducing them into neural crest lineage before they are differentiated into keratocytes. Naylor et al. conducted the two-step procedure to gain keratocyte-like cells from human iPSCs. They first cocultured the human iPSCs with bone marrow stroma cell line such as PA6 or Ms5 supplemented with FGF-2 in order to obtain neural crest lineage cells (NCCs) [33]. Subsequently, they used the NCCs either in the pellet model with KDM, which is similar to the real corneal microenvironment, or in seeding the cells on the sclera of a corneal rim slice. The gene expression of keratocyte markers from NCCs using the pellet model was 10-folds higher than the control group. The NCCs cultured on the sclera of corneal rims had even higher gene expression of keratocyte marker and reached an expression level similar to what is found in human corneal keratocytes [33]. Chan et al. cocultured human ESCs with PA6 to gain NCCs before culturing the NCCs in the pellet model with KDM, which resulted in upregulated gene expression of keratocyte-specific markers (including KERA, AQP1, ALDH3A1, CHST6, B3GNT7, and PTGDS), as well as the cells exhibiting a keratocyte-like dendritic morphology suggesting that the cells deriving from human ESCs were more sufficient as compared to iPSCs [32]. Kong et al. constructed a highly aligned 3D microfibrous scaffold similar to the physical structure of the human cornea. CSSCs were cultured on the scaffold with chemical factors including serum, insulin, FGF-2, and ascorbic acid for 2 weeks and found both an upregulation of the gene expression of keratocyte specific markers and a downregulation of fibrotic genes [66].

## 6. Perspectives

Stem cell differentiation into keratocytes has lately experienced great progression regarding the efficiency, mainly by applying either biochemical factors, physical regulation, or the combination of both. However, the keratocyte-like cells derived from differentiated stem cells are still not ideal for corneal tissue engineering as the gene expression levels of keratocyte-specific markers and proteoglycans are usually much lower than those of native keratocytes [6]. Additionally, current research lack a more refined system for evaluating whether stem cells have fully differentiated into keratocytes, which needs to be improved in the future. To generate a more efficient differentiation strategy, we propose the following possible directions in the research field:

First, novel biochemical factors need to be unravelled. Even though the recent keratocyte differentiation medium, mainly composed of FGF-2 and TGF-*β*3, is able to induce stem cell differentiation into keratocyte-like cells, it still does not reach the phenotype level as seen in the native corneal keratocytes [53]. Additionally, it is problematic to prepare KDM as it usually consists of several components, and the stem cells need at least 14 days to differentiate into keratocyte-like cells. Thus, it is of great interest to find novel and more effective biochemical factors to improve the differentiation efficacy. By comparing the expression profile of corneal tissue with other tissues to unravel what is specifically expressed in corneal tissue, important for the homeostasis of corneal tissue, might be a feasible method to discover and isolate key biochemical factors. It may also be useful to thoroughly explore the embryonic development of corneal stroma to map key factors in the process. Furthermore, cell fate is known to be regulated at multiple levels, which makes joint analysis of multiomics (epigenetic, transcriptomics, proteomics, etc.) a promising approach to better discover biochemical factors to stimulate differentiation of stem cells into keratocytes.

Secondly, when it comes to physical regulation, there are more aspects that can be considered to mimic the microenvironment of the native corneal keratocytes, such as the stiffness. A previous study has shown that stem cells will undergo lineage-specific differentiation when cultured on substrate that has similar stiffness as the native microenvironment [67]. For example, MSCs will be able to undergo superior osteogenesis capacity when cultured on 20 kPa substrate as compared to 2 kPa substrate [52]. When it comes to the corneal stroma, the physiological stiffness of the human cornea is around 24-39 kPa [68] which is much softer than the commonly used culture plate that has around 10^6^ kPa. Chen et al. found that the substrate stiffness of 25 kPa, which is similar to the natural cornea tissue, represented a positive effect on maintaining phenotype of cultured keratocytes [69]. Hence, using specific methods to adapt the stiffness of cell-culturing substrate might be a promising method to induce a more sufficient differentiation of stem cells into keratocytes. To further speculate, combining the regulation of other physical properties of scaffold material (such as the highly organized alignment of material) with the modification of stiffness may lead to synergetic effect on the differentiation of stem cells into keratocytes.

## 7. Conclusions

**Figure 3:**
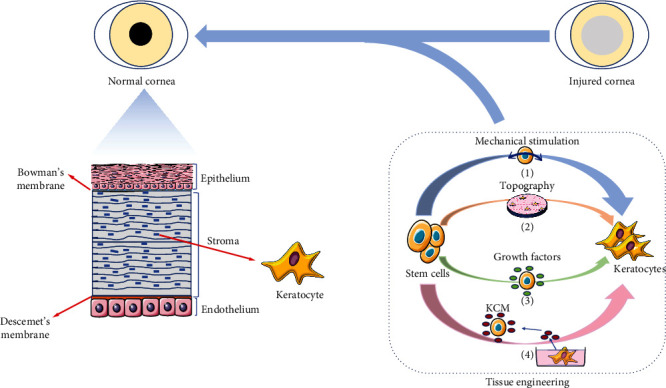
Schematic diagram summarizing the whole scope of the article. The normal cornea consists of five layers—the epithelium layer, Bowman's membrane, the stroma layer, Descemet's membrane, and the endothelium layer. We summarized the tissue engineering strategies that can make it possible to restore the damaged cornea to a normal cornea.

This review evaluates the roles of biochemical factors, physical regulation, and a combination of both in stem cell differentiation into keratocyte (Figure 3). For the biochemical approach, combinations of cytokines are used to achieve a more sufficient differentiation of stem cells into keratocytes. Regarding the physical regulation, an attempt to mimic the stress and alignment of collagen fibers of the native corneal microenvironment is achieved to improve the differentiation of stem cells into keratocytes. However, current methods to induce stem cells to differentiate into keratocytes still have their limitations as the level of keratocyte-specific genes and expression of proteoglycans is lower than that of native keratocytes. Suggested future progression is to perform attempts of finding novel effective biochemical factors either by performing in-depth analysis of factors in corneal tissue as compared to other tissues to unravel what is specifically expressed in corneal tissue or by doing a meticulous exploration of factors involved in the embryotic development of corneal stroma. A valuable aspect would be to combine the tissue-specific physical regulations in order to accomplish an experimental microenvironment that mimics the *in vivo* environment in corneal keratocytes by regulating the stiffness, topography, and other physical properties of the substrate in the experimental microenvironment.

## Figures and Tables

**Table 1:** Clinical trials of stem cells used for corneal regeneration.

ID	Title	Phase	Patients (*n*)	Stem cells	Outcome
NCT02932852	Autologous Adipose-Derived Adult Stem Cell Transplantation for Corneal Diseases	Early phase 1	12	hASCs	Vision recovery; topography; anterior segment optical coherence tomography; slit lamp observation; refraction measurement

NCT03878628	Treatment with Allogeneic Adipose-derived Mesenchymal Stem Cells in Patients with Aqueous Deficient Dry Eye Disease (MESADDE)	Early phase 1	7	hASCs	Injection site: pain, infection, bleeding; eyelid function disorder; periorbital edema; ocular discomfort; flu-like symptoms; fever; Ocular Surface Disease Index questionnaire; Schirmer's *I* test; tear osmolarity; Ocular SICCA Grading Score; HLA antibodies

NCT04932629	To Evaluate the Clinical Safety and Efficacy of Limbal Stem Cell for Treatment of Superficial Corneal Pathologies	Early phase 1	20	hCSSCs	Measurement of any ocular or systemic adverse effects; measurement of visual improvement; change in corneal light scattering

NCT01377311	The Improvement of Limbal Epithelial Culture Technique by Using Collagenase to Isolate Limbal Stem Cells	Phase 1	10	hCSSCs	Using collagenase to isolate limbal stem cells and improve the technique of ex vivo expansion of limbal stem cells for the treatment

NCT03295292	Limbus-derived Stem Cells for Prevention of Postoperative Corneal Haze	Phase 1	15	hCSSCs	Maintenance of preoperative best spectacle-corrected visual acuity; efficacy in reducing corneal light scatter using Scheimpflug imaging

NCT02948023	Stem Cells Therapy for Corneal Blindness (ExCell)	Phase 1	100	hCSSCs	Ocular or systemic adverse effects; visual improvement after treatment

NCT04484402	Treatment of Patients with Inflammatory-dystrophicDiseases of the Cornea Using Autologous Stem Cells	Phase 1 Phase 2	25	hASCs/hCSSCs	Number of cured patients, patients with treatment-related adverse events

NCT01562002	Safety Study of Stem Cell Transplant to Treat Limbus Insufficiency Syndrome	Phase 1 Phase 2	27	hBMSCs/hCSSCs	Viability and safety of mesenchymal stem cell transplant; absence of complications in pre- and perisurgical implantation; improvement of 2 lines in best-corrected visual acuity

NCT02148016	Corneal Epithelium Repair and Therapy Using Autologous Limbal Stem Cell Transplantation	Phase 1 Phase 2	30	hCSSCs	Composite measure of visual function in eyes treated for corneal ocular surface disease; composite measure of visual function in eyes after photorefractive keratectomy; incidence of transparency of the cornea; postoperative complications

NCT02592330	Limbal Stem Cell Deficiency (LSCD) Treatment with Cultivated Stem Cell (CALEC) Graft (CALEC)	Phase 1 Phase 2	17	hCSSCs	The occurrence of ocular infection, corneal perforation, graft detachment ≥ 50%, and adverse events and their relationship to the study intervention; obtaining cell growth and maintaining cell viability; avoiding culture contamination; improvement in corneal surface integrity; decrease in neovascularization; decrease in subject symptoms

NCT02318485	Limbal Epithelial Stem Cell Transplantation: A Phase II Multicenter Trial (MLEC)	Phase 2	60	hCSSCs	Visual acuity; presence of persistent epithelial defects; presence of corneal conjunctivalization; change in corneal vascularization; pain; photophobia; rejection

NCT04615455	Mesenchymal Stem Cell Therapy of Dry Eye Disease in Patients with Sjögren's Syndrome (AMASS)	Phase 2	40	hASCs	OSDI; noninvasive keratography tear break-up time (NIKBUT); tear meniscus height (TMH); Schirmer's *I* test; tear osmolarity; Oxford scale; HLA antibodies

Source of data: all comes from https://clinicaltrials.gov.

**Table 2:** Biochemical stimulation induces keratocyte differentiation.

Stem cells	Treatments	Effects
hMSCs	KCM	Spindle shaped; upregulated gene expression of keratocyte markers (KERA and ALDH1A1); downregulated expression of fibrotic marker (*α*-SMA) [27]
	KCM+AM	Dendritic or stellate morphology; upregulated gene expression of keratocyte markers (KERA and ALDH1A1); downregulated expression of fibrotic marker (*α*-SMA) [27]
	KCM+IGFBP2	Upregulated gene expression of keratocyte markers (KERA, LUM, and ALDH1A1) and downregulated expression of fibrotic marker (*α*-SMA) [38]
hCSSCs	FGF-2	Keratocyte-like morphology; barely no expression of myofibroblastic marker (*α*-SMA); increased protein and gene expression of keratocytes (KERA, LUM, ALDH3A1); decreased protein and gene expression of stem cells (Paired Box Gene 6 (Pax6), N-cadherin) [57]
	TGF-*β*3	Upregulated gene expression of keratocyte markers (KERA, beta-1,3-N-acetylglucosaminyltransferase 7 (B3GnT7), CHST6) [53]
	FGF-2+TGF-*β*3	Upregulated gene expression of keratocyte markers (KERA, B3GnT7, CHST6) [53]
ASCs	KDM^1^+RA	Upregulated gene expression of keratocyte markers (KERA, ALDH3A1, LUM, decorin); higher acid sulfated glycosaminoglycans' secretion; decreased expression of fibrotic marker (*α*-SMA) [2]
LBSCs	KDM^2^	Decreased expression of the stem cell genes (adenosine triphosphate-binding cassette G2 (ABCG2) and Nestin); increased gene expression of keratocyte markers (ALDH3A1, aquaporin1 (AQP1), KERA, and prostaglandin D2 synthase); secretion of ECM [30]
hPDLSCs	KDM^2^	Upregulated gene expression of keratocyte markers (LUM, KERA, ALDH3A1, ALDH1A1, COL I, COL V, COL III, COL VI) [7]

KCM: keratocyte-conditioned medium; KDM^1^: keratocyte differentiation media which consists of advanced DMEM, ascorbate-2-phosphate (A2-P), and 10 ng/ml fibroblast growth factor-2 (FGF-2); KDM^2^: keratocyte differentiation media which consists of advanced DMEM, A2-P, 10 ng/ml FGF-2, and 0.1 ng/ml transforming growth factor-*β*3 (TGF-*β*3); AM: amniotic membrane; RA: retinoic acid; IGFBP2: insulin-like growth factor binding protein 2.

**Table 3:** Systematic regulation induces keratocyte differentiation.

Stem cells	Systematic regulation	Findings
hESCs	Stem cells cocultured with mouse PA6 fibroblasts in serum-free medium containing ascorbate in order to generate NCCs. Subsequently, NCCs were cultured in the pellet model supplemented with KDM	Upregulated gene expression of keratocyte markers (AQP1, B3GNT7, PTDGS, and ALDH3A1); increased secretion of corneal-specific proteoglycan [32]
hiPSCs	Stem cells cocultured with bone marrow stroma cell line such as PA6 or MS5, supplemented with FGF-2 to generate NCCs. Subsequently, NCCs were cultured in the pellet model supplemented with KDM	Upregulated gene and protein expression of keratocyte markers (ALDH3A1, KERA, PTDGS, AQP1, CHST6) [33]
	Stem cells were seeded onto the sclera of corneal rim slice (specific niche including both the physical and biochemical regulations)	Keratocyte-like morphology; upregulated gene expression of keratocyte markers (ALDH3A1, KERA, PTDGS, AQP1, CHST6) [33]
hPDLSCs	Stem cells cultured in the pellet model supplemented with CSK induction media	Keratocyte-like morphology; upregulated gene expression of keratocyte markers (CD34, ALDH3A1, KERA, LUM, CHST6, B3GNT7, Collagen Type VIII Alpha 2 Chain (Col8A2)) [6]
	Stem cells cultured in the pellet model on human amnion stroma (specific niche including both the physical and biochemical regulations)	Keratocyte-like morphology; suppression of fibroblast genes (*α*-SMA); upregulated gene expression of keratocyte markers (CD34, ALDH3A1, KERA, LUM, CHST6, B3GNT7, Col8A2) [6]
	Stem cells cultured on porcine corneal stroma (specific niche including both the physical and biochemical regulations)	Presence of keratocyte gene expression (CD34, ALDH3A1, KERA, LUM, CHST6, B3GNT7, Col8A2); negligible fibroblast gene expression (*α*-SMA) [6]
ASCs	Stem cells cultured on fibrin gel supplemented with KDM	Presence of the stroma-specific ECM molecules; keratocyte-like cells; presence of less consistent expression of both KERA and keratan sulfate at protein and mRNA level [1]
	Stem cells cultured in the pellet model supplemented with KDM	Presence of the stroma-specific ECM molecules; keratocyte-like cells; presence of more consistent expression of both KERA and keratan sulfate at protein and mRNA level [1]
hCCSCs	Stem cells cultured on fibrin gel supplemented with KDM	Lower level of KERA mRNA compared with that cultured in pellet; presence of the stroma-specific ECM molecules [1]
	Stem cells were cultured on highly aligned 3D gel MA hydrogel scaffold with the supplementation of chemical factors (serum, insulin, FGF-2, and ascorbic acid)	Upregulated expression of keratocytes' genes (KERA, AQP1, and ALDH3A1); Downregulated expression of fibroblastic genes (*α*-SMA) [66]
hMSCs	Stem cells were transplanted into mice's cornea	Upregulated expression of keratocytes' mRNA (KERA and LUM) [36]

NCCs: neural crest cells; KDM: keratocyte differentiation media which consist of advanced DMEM, ascorbate-2-phosphate (A2-P), and 10 ng/ml fibroblast growth factor-2 (FGF-2); CSK: corneal stroma keratocyte induction media which consist of DMEM/F12, insulin-selenate-transferrin, ascorbate-2-phosphate (A2-P), 20 ng/ml fibroblast growth factor-2 (FGF-2), and 0.1 ng/ml transforming growth factor-*β*3 (TGF-*β*3).
